# Benchmarking bias in embeddings of healthcare AI models: using SD-WEAT for detection and measurement across sensitive populations

**DOI:** 10.1186/s12911-025-03102-8

**Published:** 2025-07-10

**Authors:** Magnus Gray, Leihong Wu

**Affiliations:** Division of Bioinformatics and Biostatistics, National Center for Toxicological Research, U.S. FDA, 3900 NCTR Rd, Jefferson, AR 72079 USA

**Keywords:** Bias, Bias measurement, Natural Language processing, Language models, Artificial intelligence, Input embeddings, Healthcare, Medicine

## Abstract

**Background:**

Artificial intelligence (AI) has been shown to exhibit and perpetuate human biases; recent research efforts have focused on measuring bias within the input embeddings of AI language models, especially with non-binary classifications that are common in medicine and healthcare scenarios. For instance, ethnicity-linked terms might include categories such as Asian, Black, Hispanic, and White, complicating the definition of – traditionally binary – attribute groups. In this study, we aimed to develop a new framework to detect and measure inherent medical biases based on SD-WEAT (Standard Deviation - Word Embedding Association Test). Compared to its predecessor, WEAT, SD-WEAT was able to measure bias among multi-level attribute groups common in the field of medicine, such as age, race, and region.

**Methods:**

We constructed a collection of medicine-based benchmarks that can be used to detect and measure biases among sex, ethnicities, and medical conditions. Then, we evaluated a collection of language models, including GloVe, BERT, LegalBERT, BioBERT, GPT-2, and BioGPT, and determined which had potential undesirable or desirable healthcare biases.

**Results:**

With the presented framework, we were able to detect and measure a significant presence of bias among gender-linked (*P* < 0.01) and ethnicity-linked (*P* < 0.01) medical conditions for a biomedicine-focused language model (e.g., BioBERT) compared to general BERT models. In addition, we demonstrated that SD-WEAT was capable of simultaneously handling multiple attribute groups, detecting and measuring bias among a collection of ethnicity-linked medical conditions and multiple ethnic/racial groups.

**Conclusions:**

To conclude, we presented an AI bias measurement framework, based on SD-WEAT. This framework provided a promising approach to detect and measure biases in language models that have been applied in biomedical/healthcare text analysis.

**Supplementary Information:**

The online version contains supplementary material available at 10.1186/s12911-025-03102-8.

## Background

Artificial intelligence (AI) is set to revolutionize the world, with rapid advances in this technology being applied to countless industries. As seen with the rise in popularity of large language models (LLMs) such as GPT-4 [1] and Llama [2], there is a growing interest in the use of language models in fields such as healthcare [3–6]. However, these AI tools have been shown to exhibit and perpetuate human biases, causing concern about the use of these tools [7, 8]. Particularly, in LLMs bias may be introduced through how the model captures information from textual data [9]. LLMs learn from textual data by converting tokens (i.e., word pieces), words, or sentences, into numerical vector representations, and thus, they may capture societal biases based on a text’s association with another.

In medical research, there are many topics or areas where bias in an AI model may be a concern. For instance, in diagnostic applications, it could be beneficial for the model to be aware of well-studied disparities in medical conditions. As an example, an AI model should be knowledgeable of medical sex differences [10], such as an increased rate of breast cancer in females or an increased rate of color blindness in males. Conversely, the model should make no such association between a sex and conditions where there is no significant sex disparity. Bias quantification is important to help determine which biases have potential to critically impact a model’s fairness towards sensitive populations. Thus, with a lack of bias benchmarks in the area, there is a need to develop such benchmark datasets for bias measurement tools such as the Word Embedding Association Test (WEAT) [11] in order to assess biases more relevant to medical models.

Regarding bias measurement, several methods have been developed to quantify and measure bias within the input embeddings of LLMs, including WEAT [11] and its extension the Sentence Encoder Association Test (SEAT) [12]. WEAT was developed in 2017 to assess bias within the semantic representations of words, or word embeddings. This method works by comparing the relative similarity of two sets of target words (e.g., math and art terms) and two sets of attribute words (e.g., male and female terms). Previous research has shown that biases in word embeddings can propagate to downstream tasks, including clinical prediction tasks [13], and thus, WEAT can play a critical role in assessing the lexical-level biases that may influence model prediction in tasks such as named entity recognition and text classification. SEAT extends WEAT to work with sentence embeddings rather than word embeddings. While both methods can be used to determine the differences in bias between groups, they have intrinsic limitations which restrict their applications. Specifically, WEAT and SEAT both rely on binary groups of words/sentences, that is, they can only measure biases among two different demographic groups. While this may work for demographic traits such as sex/gender (which is typically split into two groups: male and female), these methods are restricted when it comes to demographic traits such as race or ethnicity, which cannot be cleanly split into two groups. Moreover, both methods utilize incomplete, arbitrary-sized word/sentence groups, which may have unintended effects on their bias measurements.

Recently, we proposed standard deviation (SD)-WEAT as a more robust measure of bias in input embeddings [14]. SD-WEAT is a modification of WEAT [11] that operates by computing the SD of the effect sizes (bias scores) for multiple permutations of the WEAT benchmark datasets. This method has been shown to address the following limitations of WEAT: (1) the use of pre-defined, fixed-sized attribute word sets and (2) the necessary binary classification of attribute words. Moreover, SD-WEAT has been shown to produce more reliable and consistent measures of bias over multiple training iterations of language models [14]. The advantages of SD-WEAT are invaluable in the field of healthcare. With SD-WEAT’s random generation of attribute term sets, it enables bias assessment over multiple categories. As a result, SD-WEAT should be able to measure bias with multi-level attribute groups such as age, race, and region, making it applicable to many areas in healthcare. This, along with producing more consistent bias measurements, makes SD-WEAT more useful for assessing bias in language models for healthcare applications.

In this study, we first constructed several new benchmark datasets to measure biases related to the field of healthcare, hypothesizing that benchmarks focusing on medical conditions with sex or ethnic disparities would aid in revealing medical biases in AI models. Then, we leveraged SD-WEAT—which allows for bias assessment over multiple groups without controlling for group size—to assess these biases in several existing AI language models. It should be noted that not all biases are harmful, and in fact, one may prefer certain biases present within their AI language model (e.g., in diagnostic applications, it could be preferable to have a bias between gender terms and sex-specific medical conditions). Thus, the benchmark datasets developed in this study serve as a method for assessing healthcare-related biases, and it should be up to healthcare professionals to determine if these biases are favorable or unfavorable in their specific use-case.

## Methods

### Constructing benchmarks for healthcare bias evaluation

In the development of the benchmark datasets, we utilized government or peer-reviewed articles to source the words included in the target sets. One requirement was that the selected term had to be included in the Global Vectors for Word Representation (GloVe) [15] dictionary. In other words, if the medical term was not recognized by the GloVe model (our baseline model), it would not be considered to ensure that the embedding result is valid and comparable.

Two primary types of benchmark datasets were developed to assess biases relevant to the field of healthcare. The first type (i.e., gender-linked benchmarks) looked at the associations between gender and medical conditions / adverse drug reactions, while the second type (i.e., ethnicity-linked benchmarks) looked at the associations between ethnicity-linked terms and medical conditions. For comparative analysis, a hybrid benchmark that looked at the associations between ethnicity-linked terms and gender-linked medical conditions was also formed as a type of negative control. In total, seven benchmark datasets were collated, Table 1 in the Results section documents these benchmarks including target and attribute sets.

It should be noted that, in some of these benchmarks, there might be terms that overlap between the attribute categories. For instance, breast cancer affects both males and females, but because it occurs in women in 99% or more cases, the term “breast cancer” was placed in the “female” category in the G-1 benchmark (discussed in the next section). Thus, a high bias score in this benchmark may indicate that the model makes the typical association between women and breast cancer. In case one wants to avoid overlooking the possibility of men having breast cancer, it would be desirable to have a low bias score on this benchmark.

### Gender-linked benchmarks

For the gender-linked benchmarks, the gender-term attribute sets were derived from the original WEAT study, borrowing the attribute terms utilized in WEAT-7 [11]. For reference, WEAT-7 is a benchmark for measuring gender bias among math and arts, using terms like “male” and “female” to represent the gender attribute groups.

With the assigned gender groups, three datasets with different medical-condition target terms were defined based on their level of association between the gender attribute words. In the first dataset (G-1), the selected medical conditions are nearly exclusive to one sex, having at least a prevalence ratio of 99:1 for the respective gender. In the second dataset (G-2), the previous target sets were expanded with medical conditions that have at least a prevalence ratio of 3:1 for the respective gender. Finally, in the third dataset (G-3), the selected medical conditions were typically unrelated to one specific sex, having a prevalence ratio of 1.25:1 or less among the sexes. In the construction of the G-1 and G-2 datasets, we started by finding an article on sex differences in medicine from Wikipedia [16]. Then, we sourced various government and peer-reviewed articles that show that the selected conditions have at least a 99:1 or 3:1 prevalence ratio among sexes, respectively. Conversely, for G-3, we found a generic list of medical conditions, excluded the diseases from G-1 and G-2, and searched Google with queries such as “male vs. female rate of [condition]”. Then, we looked at articles that reported this prevalence ratio, and if it was less than 1.25:1, it was included in the dataset.

Besides the medical condition terms, a fourth gender-linked benchmark dataset was formed based on a study that analyzed sex differences among the adverse reactions [17]. In this dataset (G-4), the selected adverse drug reactions were found to be significantly linked with one gender. More specifically, we found the top ten (non-repeating) adverse drug reactions for each gender based on the logarithmic reporting odds ratio (ROR), with logarithmic RORs greater than 6.0 for female-linked reactions and less than − 6.0 for male-linked reactions.

### Ethnicity-linked benchmarks

For the ethnicity-linked benchmarks, we constructed two datasets, with the first dataset in the traditional binary format and the second dataset including multiple classes. In the development of the first dataset (E-1), we looked at two well-studied cases of hereditary genetic conditions that resulted from population bottlenecks. Particularly, the medical-condition target sets are composed of documented Finnish and Ashkenazi Jewish hereditary conditions, with the ethnicity-term attribute sets based on these two ethnicities. In the development of the E-1 dataset, we started by finding articles about Finnish and Ashkenazi Jewish heritage diseases from Wikipedia [18, 19] and sources that labeled these heritage diseases [20, 21]. Terms that were overly specific or included specific types (e.g., “amyloidosis, Finnish type” or “diarrhea 1, secretory chloride, congenital”) were excluded. Furthermore, due to the rarity of some of these medical conditions, many terms were excluded for not being found in the GloVe model’s dictionary, resulting in relatively small groups of target terms. The attribute sets for this benchmark were formed based on terms related to the nationalities and ethnic groups most affected by the conditions (e.g., “Finn” or “Finnish” for the Finnish heritage diseases and “Ashkenazi” or “Jewish” for the Ashkenazi Jewish heritage diseases).

For the second ethnicity-linked dataset (E-2), we utilized a report [22] that found different prevalence rates of various medical conditions across Black, Hispanic, and White populations. This study compares the disparities in prevalence rates between White-Non-Hispanic and Black populations as well as White-Non-Hispanic and Hispanic populations. If the Black disparity rate was greater than the Hispanic disparity rate for a medical condition, it was placed in the first target set (and vice versa with the second target set). Terms were excluded if the Black and Hispanic disparity rates were equal or if the condition was more prevalent in the White-Non-Hispanic population. The attributes for this benchmark were formed based on common terms related to the ethnic/racial groups (e.g., “African”, “Hispanic”, “Caucasian” for the Black, Hispanic, and White populations, respectively). The terms included within this benchmark are specific to the United States, as the referenced study [22] was based in this region.

### Hybrid benchmark dataset

Finally, for a hybrid analysis, a dataset was constructed based on the gender and ethnicity-linked benchmarks. In this dataset (H-1), the near-exclusive, sex-specific medical conditions of the first gender benchmark (G-1) served as the target sets, while the Black, Hispanic, and White ethnicity-linked terms of the second ethnicity-linked benchmark (E-2) were utilized as the attribute sets. Since the target terms should be stereotypically aligned with a sex rather than an ethnicity, this dataset serves as a benchmark to analyze the language understanding capability of models. In other words, if a language model was found to be significantly biased on this benchmark, then it may not have a strong understanding of the words present in this dataset.

### Language models

In this study, we evaluated six language models, for which bias can be evaluated though their embedding spaces. Particularly, we evaluated the following models: (1) GloVe [15], (2) Bidirectional Encoder Representations from Transformers (BERT) [23], (3) LegalBERT [24], and (4) BioBERT [25], Generative Pretrained Transformer 2 (GPT-2) [26], and BioGPT [27].

GloVe is an unsupervised learning algorithm for obtaining vector representations for words, trained on aggregated word-word cooccurrence statistics from a large corpus [15]. In this study, we follow suit with the original WEAT study and utilized the GloVe model “glove.840B.300d” from the Stanford NLP Group [15], which was trained on 840 billion tokens from Common Crawl data, has a vocab size of 2.2 million, and generated 300-dimension vectors. This general language model acts as a baseline.

With transformer-based language models being the current standard (as seen with GPT-4 and Llama-2), five such models were also evaluated, including three encoder models (BERT, LegalBERT, and BioBERT) and two decoder models (GPT and BioGPT). Regarding the encoder models, first, BERT is language model that was pretrained using the Toronto Book Corpus and English Wikipedia datasets with the tasks of masked language model and next sentence prediction [23]. Compared to GloVe which generates context-free representations, BERT utilizes the context on either side of a word to generate that word’s representation. This model serves as a baseline for the other transformer-based language models. Second, LegalBERT, borrowing BERT’s architecture, is a language model that was pre-trained on legal data, including legislation, court cases, and contracts [24]. Since laws and regulations play a role in the world of healthcare, this model is relevant to this study. However, since legal writings typically aim to be fair and unbiased, it is expected that this model will produce few significant (biased) results. Third, BioBERT, borrowing both BERT’s architecture and training datasets, is a language model that continued to pretrain BERT with a large collection of PubMed abstracts, resulting in a model that performs well on biomedical tasks [25]. Since this model is familiar with many medical and healthcare concepts, it was expected that this model will show significant associations between the gender or ethnicity-linked terms and medical conditions.

Regarding the decoder models, GPT-2 and BioGPT were selected due to their widespread use and relevance in general and biomedical language processing, respectively. GPT-2 is an autoregressive language model trained to predict the next token in a sequence, allowing it to generate coherent and contextually appropriate text [26]. Unlike encoder models that rely on masked token predictions, GPT models generate representations dynamically based on left-to-right context, which may influence the way bias manifests in their embeddings. BioGPT, an adaptation of GPT-2 specifically trained on biomedical literature, offers domain-specific knowledge similar to BioBERT but with the autoregressive architecture of GPT-2 [27]. This model was pretrained using millions of PubMed articles and abstracts, making it a strong candidate for evaluating bias in biomedical discourse. Due to the nature of its training data, it is hypothesized that this model may exhibit similar patterns of bias to BioBERT. Altogether, for these five transformer-based language models, we used “google-bert/bert-base-cased” [28], “nlpaueb/legal-bert-base-uncased” [29], “dmis-lab/biobert-v1.1” [30], “openai-community/gpt2” [31], and “microsoft/biogpt” [32] from Hugging Face, respectively.

While there are many other language models that are relevant to our study, such as the biomedical ClinicalBERT or MedBERT models, the main focus of this study is to provide tools and methods for assessing healthcare related biases. Thus, this analysis focuses on showcasing the applications of the developed tools and laying the groundwork for future investigations, such as a comprehensive analysis of different models.

### SD-WEAT process

In SD-WEAT, for each of the benchmark datasets, the attribute words were pooled together into one list, and then 100 WEAT tests were constructed, pulling four words from said list to form two new attribute sets (of two words each). Next, the language models were used to generate embeddings for each of the words. To ensure a more wholistic embedding of multi-token medical terms, the mean pooling method [33] is utilized, averaging the embeddings of all sub-word tokens. Finally, the SD-WEAT bias scores and *P* values were calculated. See Additional File 1 for equations and more information regarding SD-WEAT.

### WEAT process

In WEAT, the benchmark datasets follow the standard binary format with two target sets and two attribute sets. For example, for the H-1 benchmark, the two target sets are composed of male-linked and female-linked medical conditions, respectively. Similarly, the two attribute sets are composed of pairs of ethnicity-linked terms (i.e., Black and Hispanic, Black and White, etc.). After generating embeddings for each word with the language models and averaging the embeddings for all sub-word tokens with the mean pooling method [33], bias is measured between these pairs of target and attribute sets with the default WEAT effect size and P value formulae. See the original WEAT paper [11] for more information regarding WEAT.

### Detecting attribute word importance

In an effort to analyze the importance of the attribute terms, some of the SD-WEAT experiments were repeated, but with different combinations of attribute words, in order to add noise to the results. Specifically, the following sixteen terms were added to the attributes of the first three gender-linked benchmarks (i.e., G-1, G-2, and G-3): *rock*,* dirt*,* sand*,* clay*,* sky*,* cloud*,* water*,* air*,* car*,* bicycle*,* train*,* plane*,* pen*,* paper*,* book*,* laptop*.

The new words refer to non-living, inanimate objects, in order to avoid any conflict with objects that may have some interaction with the target medical conditions. However, it is possible that, through stereotypical relationships between one gender and the above words, some of these new words may have indirect associations with gender-linked medical conditions.

For this investigation, five hundred WEAT tests were generated, allowing for an extensive analysis of the importance of the attribute terms. The impact of the attribute words on the WEAT scores was then quantified, by taking the average effect size of each test where the word appeared in either the first or second attribute set. For tests where the attribute word appeared in Attribute 1, the average of the effect sizes was used to calculate its impact on or association with Target 1 (same between Attribute 2 and Target 2).

## Results

### Healthcare bias benchmarks

Table 1 summarizes each of the developed benchmarks; the complete datasets are available in Additional File 2. In Table 1, short descriptions of the datasets are provided, as well as examples of their target (T1 and T2) and attribute (A1 and A2) sets. Moreover, the number of terms included in each set are given, and sources related to the target terms’ inclusion are provided.

**Table 1:** Summary of healthcare bias benchmarks^a^

Name	Description	Target and attribute terms (Example)	Term count	References
G-1	Sex disparities in medical conditions with a sex ratio of ≥ 99:1	**T1**: prostate cancer, testicular cancer **T2**: ovarian cancer, breast cancer **A1**: male, man **A2**: female, woman	**T**: 6 × 2 **A**: 8 × 2	**T1**: [34–39] **T2**: [40–45]
G-2	Sex disparities in medical conditions with a sex ratio of ≥ 3:1	**T1**: prostate cancer, color blindness **T2**: ovarian cancer, osteoporosis **A1**: male, man **A2**: female, woman	**T**: 11 × 2 **A**: 8 × 2	**T1**: [34–39, 46–50] **T2**: [40–45, 51–54]
G-3	Sex disparities in medical conditions with a sex ratio of ≤ 1.25:1	**T1**: Hodgkin lymphoma, meningitis **T2**: cellulitis, hemorrhoids **A1**: male, man **A2**: female, woman	**T**: 5 × 2 **A**: 8 × 2	**T1**: [55–59] **T2**: [60–64]
G-4	Sex differences in adverse drug reactions	**T1**: erectile dysfunction, incisional hernia **T2**: platelet disorder, motor dysfunction **A1**: male, man **A2**: female, woman	**T**: 10 × 2 **A**: 8 × 2	**T1**: [17] **T2**: [17]
E-1	Ethnic disparities in Finnish and Ashkenazi Jewish hereditary genetic conditions	**T1**: Cohen syndrome, Salla disease **T2**: Bloom syndrome, Tay-Sachs disease **A1**: Finnish, Finn **A2**: Ashkenazi, Jewish	**T**: 5 × 2 **A**: 7 × 2	**T1**: [20] **T2**: [21]
E-2	Racial disparities in medical condition prevalence	**T1**: alopecia areata, sickle-cell anemia **T2**, hypertriglyceridemia, vitiligo **A**: African, Black, Hispanic, Latino, Caucasian, White	**T**: 4 × 2 **A**: 12 × 1	**T1**: [22] **T2**: [22]
H-1	Hybrid benchmark combining sex-related medical conditions and racial terms (CONTROL)	**T1**: prostate cancer, testicular cancer **T2**: ovarian cancer, breast cancer **A**: African, Black, Hispanic, Latino, Caucasian, White	**T**: 6 × 2 **A**: 12 × 1	**T1**: [34–39] **T2**: [40–45]

^a^Table abbreviations: G – Gender Linked; E – Ethnicity Linked; H – Hybrid; T – Target; A – Attribute

### SD-WEAT results

Table 2 summarizes the SD-WEAT results obtained for each of the developed benchmark datasets. For each model and benchmark, the table contains the SD-WEAT scores, with values significant at *P* < 0.05 marked with an asterisk (*). *P* values are provided underneath the SD-WEAT scores in parentheses.

**Table 2:** SD-WEAT scores of model embeddings on developed benchmarks (*P* Values).^a, b^

	G-1	G-2	G-3	G-4	E-1	E-2	H-1
**GloVe**	0.900* (0.006)	0.833* (< 0.001)	0.553 (0.441)	0.255 (0.999)	0.659 (0.287)	0.556 (0.998)	0.563 (0.888)
**BERT**	0.537 (0.188)	0.555* (< 0.001)	0.938 (0.113)	0.264 (0.968)	0.343 (0.112)	0.637 (0.284)	0.700* (< 0.001)
**LegalBERT**	0.400* (0.005)	0.200 (0.700)	0.749 (0.146)	0.675 (0.116)	0.348* (< 0.001)	0.663* (< 0.001)	0.302 (0.954)
**BioBERT**	0.727* (0.003)	0.608* (< 0.001)	0.558 (0.956)	0.603 (0.094)	0.844* (0.004)	0.868* (< 0.001)	0.652 (0.339)
**GPT-2**	0.449* (< 0.001)	0.248 (0.359)	0.588 (0.934)	0.477* (0.042)	0.626 (0.734)	0.421 (0.999)	0.394 (0.427)
**BioGPT**	0.912* (< 0.001)	0.596* (< 0.001)	0.447* (0.012)	0.341 (0.999)	0.647 (0.662)	0.823 (0.190)	0.466 (0.999)

^a^Table abbreviations: G – Gender Linked; E – Ethnicity Linked; H – Hybrid; GloVE – Global Vectors for Word Representations; BERT – Bidirectional Encoder Representations from Transformers; GPT – Generative Pretrained Transformer

^b^SD-WEAT scores marked with an asterisk (*) are significant at *P* < 0.05

### Gender-linked benchmarks

For the gender-linked benchmarks with medical condition target sets, the biomedicine-specialized models BioBERT and BioGPT produced significant SD-WEAT scores on each of the datasets containing terms with sex-disparities (i.e., G-1 and G-2). Conversely, on the G-3 benchmark, which has more general and gender-neutral medical conditions, BioBERT did not produce a significant bias score (0.558, *P* = 0.956), while BioGPT did produce a significant result (0.447, *P* = 0.012). Together, this showed that BioBERT better captured these medical conditions and their relationships with the gender terms.

For the benchmark with adverse drug reaction target sets (i.e., G-4), only one model produced a significant SD-WEAT score—GPT-2 with a *P* value of 0.042. While this may indicate that GPT-2 is aware of the connection between adverse drug reactions and gender terms, this result is likely due to noise, as this model’s biomedical variant, BioGPT produced an insignificant bias score. Along with BioGPT, BioBERT also struggles with this biomedical connection. One possible explanation for this outcome is that some adverse drug reactions are too generic and/or drug-specific (i.e., “scar” is associated with the female sex for a specific drug, not every drug) for the model to make this connection.

It should also be noted that several of the other models obtained significant results for several of these benchmarks. For instance, GloVe produced significant SD-WEAT scores for the first two benchmarks (0.900, *P* = 0.006; 0.833, *P* < 0.001), which covered sex-exclusive (99+:1) and sex-majority (3+:1) medical conditions. Many of these conditions had strong and well-documented relationship with one specific sex, and as a result, it is likely that these medical terms would occur alongside gender terms in a training corpus, boosting the likelihood that GloVe connected these concepts.

### Ethnicity-linked benchmarks

The two ethnicity-linked benchmarks served different purposes: the first examines hereditary genetic conditions among two relatively small populations (Finnish and Ashkenazi Jewish) in a binary format, while the second examines medical conditions with differing prevalence rates among three racial groups (Black, Hispanic, and White) in the first of its kind, multi-class format. In both benchmarks, LegalBERT (0.348, *P* < 0.001; 0.663, *P* < 0.001) and BioBERT (0.844, *P* = 0.004; 0.868, *P* < 0.001) produced significant SD-WEAT scores. This could indicate that these models better capture these medical terms and their connections to the ethnicity-linked terms. The medical conditions included within these benchmarks are generally more specific and less prominent in common language. For instance, these benchmarks include terms like “cartilage hair hypoplasia” and “hidradenitis suppurativa,” which are far less common than terms like “breast cancer” and “testicular cancer.” Thus, this is one possible reason that these terms result in less significant results, especially for the general-purpose language models. Furthermore, the results for the multi-class benchmark demonstrate that SD-WEAT can efficiently detect potential healthcare biases among multiple sensitive populations, although additional analyses that break-down the groups into pairs may be valuable for discovering the critical sources of bias.

### Hybrid benchmark

The hybrid benchmark combines the target sets from the first gender-linked benchmark (i.e., G-1) and the attribute sets from the second ethnicity-linked benchmark (i.e., E-2). For this benchmark, BERT produced a significant SD-WEAT score (0.700, *P* < 0.001). However, since the target terms should be stereotypically aligned with a sex rather than an ethnicity, this may indicate that this model did not understand the target/attribute terms or the relationships between them.

To further demonstrate the lack of relationship between the gender-linked medical conditions and the ethnicity-linked terms, WEAT scores were calculated for each pair of ethnicity-linked terms (i.e., Black and Hispanic, Black and White, etc.) using Glove and the three BERT models. Table 3 summarizes the results obtained from this pairwise analysis. For each model and ethnicity pair, the table contains the respective WEAT scores, with *P* values provided in parentheses.

**Table 3:** Pairwise WEAT analysis on hybrid benchmark (H1).^a, b^

	Black- Hispanic	Black- White	Hispanic- Black	Hispanic- White	White- Hispanic	White- Black
**GloVe**	0.152 (0.405)	0.450 (0.232)	-0.152 (0.596)	0.020 (0.482)	-0.450 (0.769)	-0.020 (0.519)
**BERT**	0.822 (0.084)	0.553 (0.131)	-0.822 (0.917)	0.015 (0.491)	-0.553 (0.870)	-0.15 (0.510)
**LegalBERT**	0.275 (0.323)	0.240 (0.346)	-0.275 (0.679)	-0.335 (0.711)	-0.240 (0.655)	0.335 (0.290)
**BioBERT**	0.526 (0.189)	0.093 (0.442)	-0.526 (0.812)	-0.717 (0.886)	-0.093 (0.560)	0.717 (0.115)

^a^Table abbreviations: GloVE – Global Vectors for Word Representations; BERT – Bidirectional Encoder Representations from Transformers

^b^WEAT scores marked with an asterisk (*) are significant at *P* < 0.05

Based on these results, none of the WEAT scores were significant at *P* < 0.05, indicating that no model found a substantial relationship between the gender-linked medical conditions and the ethnic/racial pairs. Nonetheless, BERT produced the highest and lowest WEAT scores out of the evaluated models, including a near significant WEAT score for the “Black-Hispanic” pair (*P* = 0.084), showing similar trends to the SD-WEAT results. Overall, these results highlight the utility of SD-WEAT, which does not require such an extensive pairwise analysis to detect potential bias among multiple, non-binary classifications of attribute terms.

### Importance of attribute terms

**Fig. 1:**
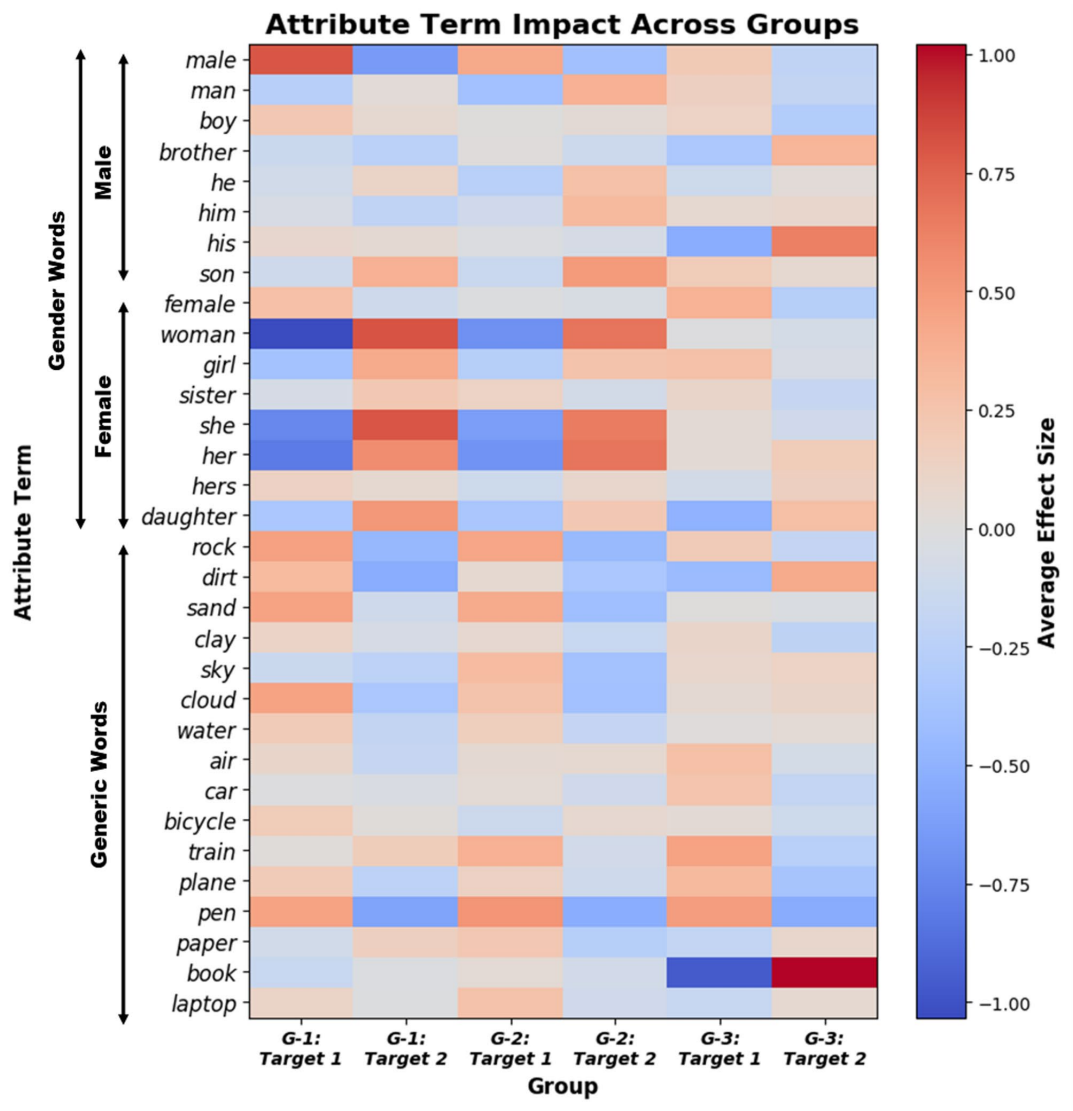
Heatmap of attribute term importance across groups for BioBERT

To further understand the impact of attribute terms (i.e., male and female terms) in the gender-linked benchmarks (i.e., G-1, G-2, and G-3), we repeated the SD-WEAT experiments for modified versions of these benchmarks that included additional, generic terms (i.e., “rock”, “sky”, etc.). Figure 1 illustrates the results of BioBERT for the analysis concerning the detection of attribute term importance. In this experiment, noise words were added to the first three gender-linked benchmarks (i.e., G-1, G-2, and G-3). The heatmap shows the average effect that the words had on the target sets of each benchmark.

Based on the results, the male and female gender terms were typically among the words that produce the highest average effect sizes for the first two benchmarks (i.e., G-1 and G-2) regarding their respective gender-linked medical conditions. For instance, looking at the groups “G-1: Target 2” and “G2: Target 2”, WEAT tests that had the words “woman”, “she”, and “her” in the second attribute sets have the highest average effect sizes on these two benchmarks, indicating that these words had a strong association with the female-related conditions included within the second target sets. However, looking at the results for G-3, which contains more general and gender-neutral medical conditions, the gender-linked terms did not have such an impact, with a more random spread of attribute words and their relationship with the target sets. Although, it can be noted, that for this benchmark, the term “book” stands out, indicating that one or more of the medical conditions had a strong connection with this term, perhaps from commonly being in book titles or frequently next to the word “book” in the training corpus. Altogether, this analysis showed that the BioBERT model strongly captures the sex differences in medicine, with greater biased associations between gender terms and sex-linked conditions than general terms and such conditions.

## Discussion

### Differences in models’ observed SD-WEAT bias scores

This study evaluated the biases of several different language models, including GloVe, BERT, LegalBERT, BioBERT, GPT-2, and BioGPT. These models have differences in their architecture, training datasets, or both, leading to diverse results on the developed benchmarks.

The baseline model, GloVe, generates word embeddings by utilizing co-occurrence statistics, or how often one word is used next to another word [15]. Thus, as seen with the G-1 and G-2 benchmarks, this model showed significant associations between words that often appear together, such as sex-specific medical conditions and gender terms. Unlike the ethnicity-linked medical conditions that are more uncommon or less studied, this model only produced biased associations for groups of terms that commonly occur with one another.

Next, the encoder models (i.e., BERT, LegalBERT, and BioBERT) generate word embeddings by utilizing the context on either side of a word to learn its meaning [23]. This, along with the different training datasets, could explain the differences in performance between BERT and GloVe. More specifically, the GloVe model utilized in this study was trained on Common Crawl data, which gives it a broader range of data sources than the BERT model trained on the Toronto Book Corpus and English Wikipedia. This, in turn, could result in the large differences in performance between GloVe and BERT.

The LegalBERT and BioBERT models utilize BERT’s architecture, but different training datasets, which may explain the difference in their performance compared to BERT. For instance, LegalBERT was trained on legal data, including legislation, court cases, and contracts [24], which typically aim to be fair and unbiased. However, this model produced significant results on the ethnicity-linked benchmarks (i.e., E-1 and E-2), which may indicate that this model was sensitive to the ethnic/racial attribute terms. Conversely, BioBERT was trained on BERT’s training datasets and a large collection of PubMed abstracts [25]. Thus, this model better captured medical and healthcare-related knowledge and the associations between both the sex and ethnicity-linked conditions and the gender and ethnicity-linked attribute terms.

Finally, the decoder models—GPT-2 and BioGPT—differ in that they generate embeddings using left-to-right autoregressive language modeling. GPT-2 was trained on a broad, web-based dataset, exposing it to a wide range of knowledge [26], which may explain its significant associations across multiple benchmarks. In contrast, BioGPT was trained specifically on biomedical texts from PubMed [27], giving it a more focused domain understanding. This specialized training likely contributed to the model’s performance on benchmarks involving sex-related medical conditions. However, given their generative nature, the biases present in these models may manifest differently compared to the encoder models, potentially amplifying or suppressing associations depending on the context of language exposure.

### Advantages of SD-WEAT

In this study, we explored the bias assessment over multi-level attribute groups, with the development of a benchmark based on ethnic/racial disparities in medicine. This benchmark, labeled E-2, was based on a study that examined the prevalence rate of various medical conditions among Black, Hispanic, and White populations. Unlike WEAT, which is confined to only using two attribute groups, SD-WEAT is capable of handling the three ethnic/racial groups at once. Additionally, with SD-WEAT’s random generation of attribute term sets, this method allows for the effective analysis of their impact on bias scores. Therefore, using SD-WEAT can facilitate the automation of bias measurement over multiple attribute groups, in addition to gaining a deeper understanding on the biased associations that models make.

### Limitations and future directions

In this study, we aimed to develop benchmarks and use SD-WEAT to examine medical biases concerning sex disparities among medical conditions and adverse drug reactions. However, we found that the connection between gender and adverse drug reactions was difficult to detect in the selected language models. Because adverse drug reactions are specific to the drug tested, some reactions are not always specific to a single sex. For instance, while the adverse reaction “scar” is linked to females for the drug heparin sodium [17], it is unlikely to be linked to this sex for every other drug. Thus, there is an issue of defining benchmarks for adverse drug reactions, and additional avenues of exploring bias in this area should be investigated.

In the future, we plan to examine how a language model’s biases can be harnessed to our advantage. Several studies have focused on debiasing, or reducing the bias of, language models [65, 66], and as a result, we may examine if we can reduce potentially harmful medical biases, such as linking sexes to general/unrelated medical conditions (i.e., benchmark G-3). Alternatively, we may formulate methods to increase the desirable biases of the models, using the developed benchmark datasets for measuring these changes. Another key component of future research is to assess the extent biases are harmful or affect a model’s performance by evaluating the AI model’s downstream performance before or after making modifications to its biases. Furthermore, due to their recent dominance, it may be beneficial to assess the biases of popular LLMs such as Llama or Mistral, in addition to exploring how model scaling impacts biases by considering different sizes of such models. Future work could also extend this study by exploring how contextual variations influence embedding biases, particularly by analyzing how a term’s associations shift across different sentences and paragraphs.

## Conclusion

To conclude, we introduced a collection of benchmarks for detecting and measuring inherent medical biases in the word embeddings of AI language models. We utilized SD-WEAT to assess the biased associations between different human sexes or ethnicities and medical conditions, showing that biomedicine-trained language models better capture the sex and ethnic disparities in medicine than more general-purpose models. Moreover, we highlighted the strengths of SD-WEAT, showing how it can handle multiple attribute groups, rather than being confined to a binary classification of attributes like its predecessor WEAT. In the future, SD-WEAT can be a useful multi-class bias quantification tool applied in regulatory science and beyond, with potential to utilize this tool alongside bias reduction techniques to further explore biases in AI language models.

## Electronic supplementary material

Below is the link to the electronic supplementary material.

Supplementary Material 1

Supplementary Material 2

Supplementary Material 3

## Data Availability

All data generated or analyzed during this study are included in this published article and its supplementary information files.

## Abbreviations

AI: Artificial intelligence
BERT: Bidirectional Encoder Representations from Transformers
GloVE: Global Vectors for Word Representations
LLM: Large language model
ROR: Reporting odds ratio
SD: Standard deviation
SEAT: Sentence Encoder Association Test
WEAT: Word Embedding Association Test
