# Supplementary material for: Benchmarking bias in embeddings of healthcare AI models: using SD-WEAT for detection and measurement across sensitive populations
Source: BMC Med Inform Decis Mak. 2025 Jul 10;25:258. doi: 10.1186/s12911-025-03102-8 (PMC12247235; doi:10.1186/s12911-025-03102-8)
Supplement: Supplementary file 1 — Supplementary Material 1 [file 12911_2025_3102_MOESM1_ESM.docx]

## Additional File 1: Supplementary Information on SD-WEAT

SD-WEAT scores (i.e., the bias measurement) were computed using Equation 1, where (*d*) is the effect size obtained for each WEAT test.

Equation 1: SD-WEAT Score

| $SD\_WEAT=SD\left( d_{1},d_{2},\ldots d_{100} \right)$ $where:$ $d=\frac{{mean}_{x\in X}s\left( x,A,B \right)-{mean}_{y\in Y}s\left( y,A,B \right)}{{std\_dev}_{w\epsilon X\cup Y}s\left( w,A,B \right)}$ $where:$ $s\left( w,A,B \right)={mean}_{a\in A}\cos\left( \vec{w},\vec{a} \right)-{mean}_{b\in B}\cos\left( \vec{w},\vec{b} \right)$ $where:$ $w=target word$ $A=attribute set 1$ $B=attribute set 2$ $X=target set 1$ $Y=target set 2$ |
| --- |

The significance of the SD-WEAT results were calculated through the use of a negative control experiment. In this negative control, 10,000 new tests were constructed, pulling four words from a large dictionary of words to form two new attribute sets (of two words each). This creates 100 groups of 100 tests, allowing us to analyze variance across groups and compute Z-scores and *P* values for the SD-WEAT scores. The Z-scores were calculated using Equation 2, where *x* is the SD-WEAT score, while *μ* and *σ* are the average and SD of the SDs for the 100 groups of 100 effect sizes in the negative control, respectively. Since WEAT uses a one-sided, right-tailed test, *P* values were calculated from the Z-scores with the right-tailed methodology.

Equation 2: SD-WEAT Significance Calculation

| $Z=\frac{x-\mu}{\sigma}$ $where:$ $x=SD\_WEAT$ $x\_control=({SD\left( d_{1},d_{2},\ldots d_{100} \right)}_{1},\ldots{SD\left( d_{1},d_{2},\ldots d_{100} \right)}_{100})$ $\mu=mean(x\_control)$ $\sigma=SD(x\_control)$ |
| --- |
